# Test-retest reliability and measurement error of the pain interference questionnaire for cerebral palsy and the fear of pain questionnaire adapted for cerebral palsy

**DOI:** 10.1186/s41687-026-01129-7

**Published:** 2026-06-17

**Authors:** Meredith Grace Smith, Nadine Smith, Georgia McKenzie, Adrienne Harvey

**Affiliations:** 1 School of Allied Health and Human Performance, Adelaide University, Adelaide, Australia; 2 Women‘s and Children‘s Health Network, North Adelaide, Australia; 3 Kids Rehab, Perth Children‘s Hospital, Perth, Australia; 4 University of Western Australia, Perth, Australia; 5 Young Adult Complex Disability Service, St Vincent‘s Hospital, Melbourne, Australia; 6 Neurodisability and Rehabilitation, Murdoch Children‘s Research Institute, Melbourne, Australia; 7 School of Primary and Allied Health Care, Monash University, Melbourne, Australia

## Abstract

**Background:**

The Pain Interference Questionnaire for Cerebral Palsy (PIQ-CP) and Fear of Pain Questionnaire adapted for Cerebral Palsy (FOPQ-CP) are two patient-reported outcome measures (PROMs) developed and validated for children and young people with cerebral palsy and chronic pain, with specific adaptations to improve self-report opportunity. Test-retest reliability is an important psychometric property to ensure PROMs are used accurately to assess change in intervention and longitudinal studies. This study aimed to determine the test-retest reliability of the PIQ-CP and FOPQ-CP in children and young people with cerebral palsy.

**Methodology:**

Children and young people with CP aged 5–30 years with a history of ongoing pain were recruited from four Australian sites within a larger cross-sectional study. Participants completed the PIQ-CP or FOPQ-CP on two occasions 2–4 weeks apart. Test-retest reliability and measurement error were assessed using intraclass correlation coefficients (ICC absolute agreement), standard error of measurement (SEM) and smallest detectable change (SDC).

**Results:**

Test–retest reliability was moderate for the FOPQ-CP (Pain-related fear (subscale 1): ICC = 0.74 [0.58–0.85], SEM = 2.12, SDC = 5.88; *n* = 44; Pain-related avoidance (subscale 2): ICC = 0.62 [0.40–0.77], SEM = 4.07, SDC = 11.27; *n* = 44) and for the Pain Interference Questionnaire – Cerebral Palsy (PIQ-CP) (Pain interference: ICC = 0.71 [0.53–0.83], SEM = 6.29, SDC = 17.43; *n* = 45). The mean interval between assessments was 26.8 days.

**Conclusions:**

The PIQ-CP and FOPQ-CP have demonstrated sufficient test–retest reliability for children and young people with cerebral palsy, including for those with diverse abilities. They can now be used in clinical and research settings to prioritise self-reported pain interference and pain-related fear/avoidance in intervention studies.

**Supplementary Information:**

The online version contains supplementary material available at https://doi.org/10.1186/s41687-026-01129-7.

## Background

The Pain Interference Questionnaire for Cerebral Palsy (PIQ-CP) and the Fear of Pain Questionnaire adapted for Cerebral Palsy (FOPQ-CP) are two patient-reported outcome measures (PROMs) recently developed and validated [1–4] for children and young people with cerebral palsy, the most common cause of childhood-onset physical disability [5]. These tools were developed to address the lack of accessible measures for children and young people with diverse abilities. The PIQ-CP and FOPQ-CP use features such as pictorial supports, alternative administration options, and simplified wording and scales, all of which help improve opportunities for self-report among people with varied cognitive, communication, and movement abilities [3]. Both the PIQ-CP and the FOPQ-CP have been identified as recommended PROMs in the recently published core outcome set for the assessment of chronic pain in cerebral palsy [6], assessing the domains of pain interference (PIQ-CP), and impact of pain on emotional functioning (FOPQ-CP). More specifically, the FOPQ-CP assesses impact of pain on emotional functioning across two constructs; pain-related fear and pain-related activity avoidance [3].

While the PIQ-CP and FOPQ-CP have undergone rigorous investigation to ensure adequate content validity [1, 2, 4], structural validity [3] and internal consistency [3] in the cerebral palsy population, the test-retest reliability of the PROMs is unknown. Test-retest reliability is defined as ‘the extent to which scores for patients who have not changed are the same for repeated measurements, at different time points, under the same conditions [7, 8]. Adequate test-retest reliability ensures PROMs can be used accurately to assess change in intervention and longitudinal studies. Given the recommendation to use the PIQ-CP and FOPQ-CP as part of the chronic pain assessment in cerebral palsy core outcome set, it is important to identify the test-retest reliability of these measures. The primary aim of this study was to determine the test-retest reliability, standard error of measurement (SEM) and smallest detectable change (SDC) of the PIQ-CP and FOPQ-CP in children and young people with cerebral palsy, aged 5–30 years.

## Methods

### Study design

Test-retest reliability data was collected as part of a larger prospective cross-sectional multi-site study, guided by the COSMIN guidelines for reliability studies [9]. Ethics approval was received from the Women’s and Children’s Human Research Ethics Committee (2022/HRE10554). The study was guided by a lived experience advisory group, including two young adults with cerebral palsy and a parent of a child with cerebral palsy. The advisory group directly informed study design decisions about how to identify ongoing pain, operationalise the concept of ‘ongoing pain’ and change in pain, and timeframes for test-retest data collection.

### Participants and recruitment

Recruitment took place at four Australian sites: Novita (South Australia), Women’s and Children’s Hospital Paediatric Rehabilitation Department (South Australia), Perth Children’s Hospital Kids Rehab (Western Australia) and St Vincent’s Hospital Melbourne (Victoria) from November 2023 – November 2025. Children and young people were eligible to participate in the study if they were aged 5–30 years with a diagnosis of cerebral palsy, had a history of ongoing pain and functioned within communication function classification system (CFCS) level III or higher, meaning they could usually communicate effectively with familiar partners using any method, including verbal or augmentative and alternative communication (AAC) [10]. To determine a history of ongoing pain, children and young people were asked if they had ever experienced pain lasting longer than a few weeks, and if they answered ‘no’, they were provided with examples of common ongoing pain issues in young people as suggested by Vinkel and colleagues [11]: “some people often have muscle or joint pain after activity, some often have headaches, stomach aches or a sore back or legs. Does this sound like you?”. This was used to ensure children and young people for whom pain is a normal part of life were accurately identified [12]. Participants who were unable to self-report using the PIQ-CP or FOPQ-CP even with support were excluded. Written informed consent was obtained from young adults without cognitive impairment, caregivers of children and caregivers of young adults with cognitive impairment.

#### Participant demographics

Demographic characteristics (age, sex, communication method, gross motor ability and cognitive ability) were provided by the child/young person or their caregiver. Gross motor ability was classified using the Gross Motor Function Classification System (GMFCS), providing a 5-level rating from I (higher functional ability) to V (lower functional ability) [13].

#### Sample size

A target sample of 50 children and young people was estimated to support a target intraclass correlation coefficient (ICC) of 0.70 (two-way mixed effects model, agreement), with target confidence interval (CI) width of 0.3 (ICC). This sample size was estimated using an online simulation application developed by Mokkink and colleagues [14] (https://iriseekhout.shinyapps.io/ICCpower/).

### Measures

#### Pain interference questionnaire for cerebral palsy

The PIQ-CP is available in two formats, standard pen/paper or *Talking Mat* (available from https://doi.org/10.6084/m9.figshare.29309753). Both versions ask for a response to the same 12 items (with an optional 13th item) using the lead in phrase ‘in the past week, how has pain gotten in the way with….’. Responses are provided on a 5-point ordinal scale (0=’not at all’ to 4 = ‘a lot’), with scores totalled to produce a sum score (0–48). Higher scores indicate greater pain interference.

#### Fear of pain questionnaire adapted for CP

The FOPQ-CP is also available in two formats – standard pen/paper or *Talking Mat* (available from https://doi.org/10.6084/m9.figshare.29308421). Again, both versions ask for a response to 10 statements about how pain makes you feel, with responses provided on a 5-point ordinal scale (0=’this is not me’ to 4 = ‘this is me’). The FOPQ-CP has two distinct subscales: pain-related fear (items 1–3, 9) and pain-related activity avoidance (items 4–8, 10). Scores are totalled within each subscale to produce a sum score (pain-related fear: 0–16, pain-related activity avoidance: 0–24) with higher scores indicating greater pain-related fear and activity avoidance.

### Procedure

Data was collected with a clinician, either as part of usual clinical practice, or a stand-alone research appointment. Administration method (*Talking Mat* or pen/paper) was selected based on the decision-making tree used in Fig. 1 of Smith et al. 2025 [3]. Support was provided as needed by the clinician and/or caregiver to allow the child or young person to self-report, such as reading questions out loud or providing physical assistance. Participants were invited to repeat the assessments 2–4 weeks later. On this second occasion, assessments were completed using the same administration method as the first. Young people were also asked one question to determine if their ongoing pain may have changed since the previous assessment: “Has anything happened that would impact your pain over the last few weeks since you first did the assessment?”

Young people who completed a second assessment were provided with a gift voucher as reimbursement. Completed assessments were uploaded to REDCap electronic data capture tool hosted at University of Adelaide [15, 16]. All clinicians involved in data collection were provided with training by the author (MS).

### Data analysis

Analyses were performed in Stata 18. Demographic characteristics were descriptively summarised. Test-retest reliability was estimated for both the PIQ-CP and FOPQ-CP using intraclass correlation coefficients (ICC) (two-way mixed effects, absolute agreement, single measures) [17], standard error of measurement (SEM), smallest detectable change (SDC) and 95% CI [8]. Bland-Altman plots were included to assist in reliability interpretation. ICC values were interpreted using established categories (< 0.5 = poor, 0.5–0.75 = moderate, 0.75–0.90 = good, > 0.9 = excellent [17]. In accordance with COSMIN criteria, an ICC ≥ 0.7 was considered to indicate sufficient test re-test reliability for clinical or research use [7]. A sensitivity analysis was also completed restricted to participants who reported no change in pain status between assessments.

## Results

**Fig. 1:**
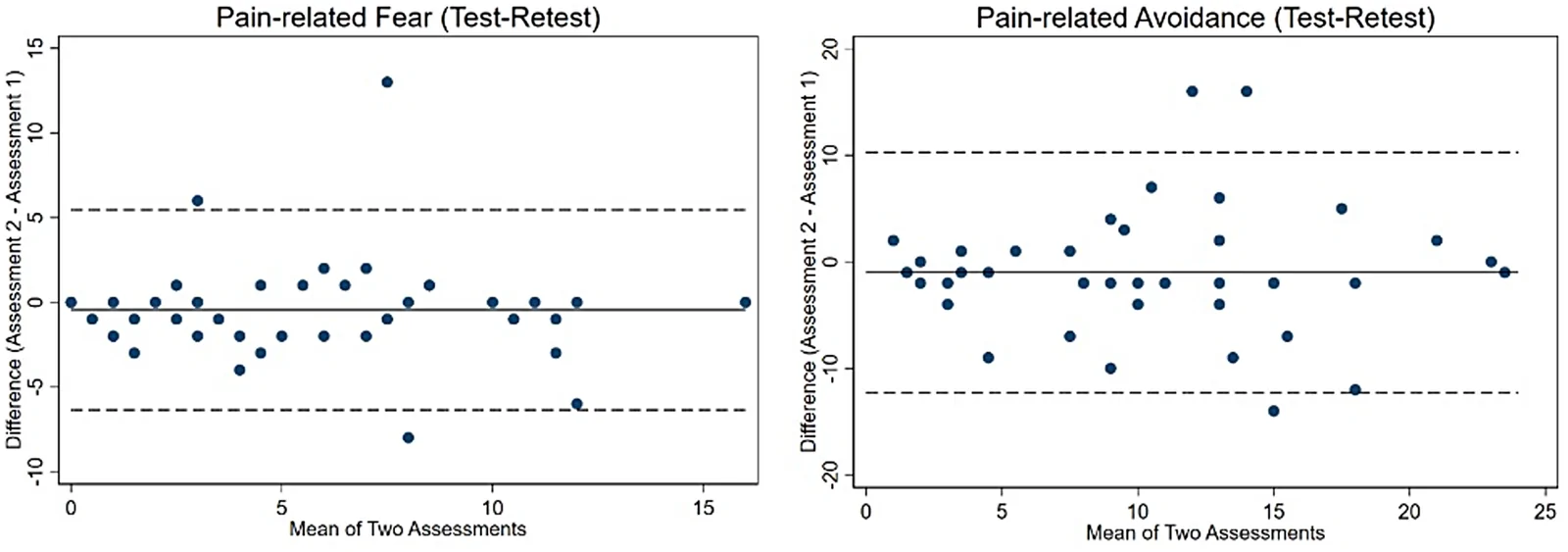
Bland altman plot of the fear of pain questionnaire adapted for cerebral palsy (pain-related fear and pain-related avoidance subscales, *n* = 44). The solid horizontal line represents the mean difference (bias). The dashed horizontal lines represent the 95% limits of agreement (LoA), calculated as mean difference ± 1.96 × SD of differences

**Fig. 2:**
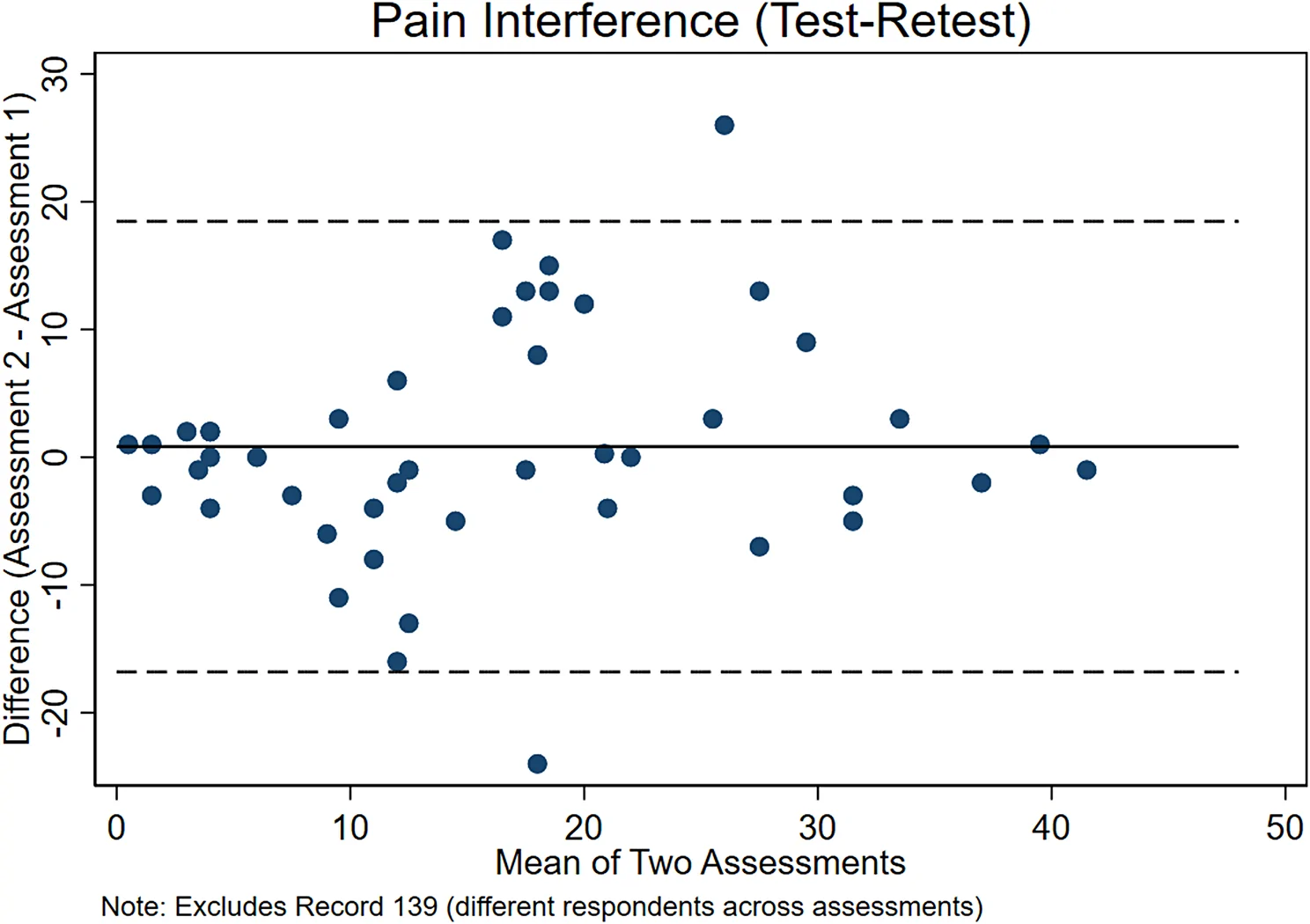
Bland altman plot of the pain interference questionnaire for cerebral palsy (*n* = 45). The solid horizontal line represents the mean difference (bias). The dashed horizontal lines represent the 95% limits of agreement (LoA), calculated as mean difference ± 1.96 × SD of differences

167 children and young people completed one assessment and 49 participants completed repeat assessments. Four were excluded from the PIQ-CP analysis and five from the FOPQ-CP due to missing data. In total, 45 PIQ-CP repeat assessments (mean age 14.2 ± 5.5 years) and 44 FOPQCP repeat assessments (mean age 13.9 ± 5.3 years) were analysed (Table 1). Descriptive statistics for baseline and repeat PIQ-CP and FOPQ-CP scores are shown in Table 2. The time between assessments was a mean of 26.8 days (SD 20.6; median 21, IQR 14–30, range 7–110) for the FOPQ-CP and 26.4 days (SD 20.5; median 19, IQR 14–28, range 7–110) for the PIQ-CP. Twenty-seven of the 44–45 participants completed the retest within the intended 2–4 week (14–28 day) window, and 11 (approximately 25%) completed it beyond 28 days. All scores demonstrated moderate test-retest reliability: Pain-related fear (FOPQ-CP subscale 1) ICC = 0.74, pain-related avoidance (FOPQ-CP subscale 2) ICC = 0.62 and pain interference (PIQ-CP) ICC = 0.71. Bland-Altman plots did not reveal systematic differences in agreement across the range of scores for pain-related fear (Fig. 1), pain-related avoidance (Fig. 1), and pain interference (Fig. 2). The smallest detectable change was 5.88 for pain-related fear (FOPQ-CP subscale 1), 11.27 for pain-related avoidance (FOPQ-CP subscale 2) and 17.43 for pain interference (PIQ-CP). A sensitivity analysis of 34 children and young people (PIQ-CP and FOPQ-CP) who reported no change in pain status between assessments (Appendix 1) demonstrated similar results to the main analysis with all ICCs remaining in the moderate range. A further sensitivity analysis restricted to participants whose retest interval was within the intended 2–4 week window (≤ 28 days) also demonstrated results consistent with the main analysis, with all ICCs remaining in the moderate range (Appendix 1).

**Table 1:** Participant demographic characteristics for participants who completed two assessments of the FOPQ-CP and PIQ-CP to assess test-retest reliability

Characteristic	FOPQ-CP (*n* = 44)	PIQ-CP (*n* = 45)
**Age, mean (SD)**	13.9 (5.3)	14.2 (5.5)
**Female, n (%)**	23 (52.3)	24 (53.3)
**GMFCS level, n (%)**		
I	9 (20.5)	9 (20.0)
II	25 (56.8)	25 (55.6)
III	4 (9.1)	4 (8.9)
IV	3 (6.8)	3 (6.7)
V	3 (6.8)	4 (8.9)
**Likely cognitive impairment, n (%)**	16 (36.4)	16 (35.6)
**Communication method, n (%)**		
Verbal	41 (93.2)	41 (91.1)
Augmentative and Alternative Communication user	3 (6.8)	4 (8.9)
**Administration method, n (%)**		
Pen/paper	33 (75.0)	34 (75.6)
Talking Mat	11 (25.0)	11 (24.4)

Abbreviations: FOPQ-CP = Fear of Pain Questionnaire adapted for Cerebral Palsy, GMFCS = Gross Motor Function Classification System, PIQ-CP = Pain Interference Questionnaire for Cerebral Palsy

**Table 2:** Test re-test reliability (FOPQ-CP and PIQ-CP) results. Mean difference calculated as score at time 2 – score at time 1 for test-retest reliability

	Test-retest reliability		
	FOPQ-CP		PIQ-CP
	Pain-related fear (*n* = 44)	Pain-related avoidance (*n* = 44)	Pain interference (*n* = 45)
**ICC (95% CI)**	0.74 (0.58–0.85)	0.62 (0.40–0.77)	0.71 (0.53–0.83)
**Standard Error of Measurement (SEM)**	2.12	4.07	6.29
**Smallest Detectable Change (SDC)**	5.88	11.27	17.43
**Mean difference (SD)**	-0.45 (3.02)	-1.00 (5.75)	0.83 (9.00)
**Limits of agreement**			
Lower (95% CI)	-6.36 (-7.91 to -4.82)	-12.28 (-15.22 to -9.33)	-16.81 (-21.36 to -12.25)
Upper (95% CI)	5.46 (3.91 to 7.00)	10.28 (7.33 to 13.22)	18.46 (13.91 to 23.02)

Abbreviations: CI = confidence interval, ICC = intraclass correlation coefficient, FOPQ-CP = Fear of Pain Questionnaire adapted for Cerebral Palsy, PIQ-CP = Pain Interference Questionnaire for Cerebral Palsy, SD = standard deviation

## Discussion

This study demonstrated sufficient test–retest reliability for two newly developed and validated PROMs for children and young people with cerebral palsy, the PIQ-CP and the FOPQ-CP, which were designed to be accessible across a wide range of cognitive, communication, and movement abilities. Reliability was adequate for the PIQ-CP and for the pain related fear subscale of the FOPQ-CP (ICC > 0.70). Although the pain related avoidance subscale showed a slightly lower ICC (0.62) than the target threshold, its 95% confidence interval (0.40–0.77) includes the target value. This suggests that the subscale may still represent a moderately stable measure over time.

Taken together with previous evidence of sufficient content validity [1, 2, 4], structural validity [3] and internal consistency [3], these results support the use of the PIQ-CP and FOPQ-CP in research and clinical settings to capture self-reported pain interference and pain related fear/avoidance from children and young people with cerebral palsy, including those with diverse abilities. This creates a new and important opportunity to include their self-reported pain information in research and clinical care focused on chronic pain management.

Both the PIQ-CP and FOPQ-CP were adapted from established paediatric pain PROMS: the PIQ-CP from a modified version of the Brief Pain Inventory [18, 19] and the FOPQ-CP from the Fear of Pain Questionnaire for Children–Short Form (FOPQ-C-SF) [20]. As the modified Brief Pain Inventory has not previously been evaluated for test–retest reliability, direct comparison with our PIQ-CP findings is not possible. In contrast, the FOPQ-C-SF has demonstrated similar test–retest reliability to our results for the FOPQ-CP, with moderate Pearson r correlations over a three month interval in children with chronic pain (fear: *r* = 0.68; avoidance: *r* = 0.52) [20]. Interestingly, both Heathcote et al. (2020) [20] and our study found lower test-retest reliability for avoidance than for fear, suggesting that avoidance may vary more over time. This is plausible, as avoidance may be more influenced by the specific activities and opportunities encountered in daily life, whereas fear-related thoughts may remain more stable [21].

This study has several strengths. First, it included participants with a wide range of cognitive and communication abilities, helping to ensure the findings are generalisable to the broader cerebral palsy population. 35% of participants had a likely cognitive impairment and were still able to complete the assessments at both time points, demonstrating the feasibility of using these tools in real-world clinical settings. In addition, the study followed the COSMIN guidelines for conducting reliability research and included a sensitivity analysis restricted to participants who reported no change in pain status during the testing period. This approach helped ensure the reliability estimates were as accurate as possible.

The study also has several limitations. The test–retest sample included only one quarter of the wider cohort and consisted solely of participants who agreed to complete two assessments. Although children and young people who use AAC were well represented in the initial assessment, they were under-represented in the test–retest reliability analysis compared with the estimated proportion of children and young people with cerebral palsy who do not use verbal communication (24.5%) [23]. This underrepresentation was likely due to the increased time and effort required to complete a second assessment using AAC. To determine if there had been changes in the constructs of interest between assessments, a brief global question was used. While this approach was intentionally chosen to reflect the constructs of interest and the known variability of pain intensity in children and young people with cerebral palsy, it may not have captured more subtle changes in pain status. Furthermore, the test re-test interval of two-four weeks and variability in retest timing may have introduced additional heterogeneity, potentially allowing for true change in pain-related experiences to influence reliability estimates despite efforts to minimise this risk. However this wider interval was selected to balance feasibility of completing the study in clinical settings and the expectation that the constructs of pain interference, pain-related fear and activity avoidance are expected to be relatively stable compared to other pain constructs such as pain intensity which vary significantly on a day-to-day basis. The final test–retest sample sizes (*n* = 44 and *n* = 45) fell slightly short of the target (*n* = 50), resulting in marginally wider ICC CIs; however, the impact on precision was minimal and not considered a major limitation. Assessors were not blinded to assessment order, but this is unlikely to have influenced responses, as the measures rely on participant self-report with minimal clinician input. The study also did not account for differences in administration methods (pen/paper versus *Talking Mat)* when calculating test–retest reliability. Because participants used the administration method most appropriate to their abilities, and many could not complete both, this is not viewed as a substantial limitation. Finally, the sample size did not allow for further exploration of how age or cognitive ability might influence the test-retest reliability of the PIQ-CP and FOPQ-CP. This could be an important area for future research.

## Conclusion

The PIQ-CP and FOPQ-CP demonstrated sufficient test–retest reliability for use with children and young people with cerebral palsy, including those with diverse cognitive, communication, and movement abilities. With valid and reliable tools established, future research can apply these tools within intervention studies to build an evidence base for effective pain interventions for children and adults with cerebral palsy.

## Supplementary Information

Below is the link to the electronic supplementary material.


Supplementary Material 1


## Data Availability

The data that support the findings of this study are available from the corresponding author, MS, upon reasonable request.

## Abbreviations

AAC: Augmentative and alternative communication
CFCS: Communication Function Classification System
CI: Confidence interval
FOPQ-C-SF: Fear of Pain Questionnaire for Children–Short Form
FOPQ-CP: Fear of Pain Questionnaire adapted for Cerebral Palsy
GMFCS: Gross Motor Function Classification System
ICC: Intraclass correlation coefficient
PIQ-CP: Pain Interference Questionnaire for Cerebral Palsy
PROMs: Patient-reported outcome measures
SDC: Smallest detectable change
SEM: Standard error of measurement
