# Supplementary material for: Test-retest reliability and measurement error of the pain interference questionnaire for cerebral palsy and the fear of pain questionnaire adapted for cerebral palsy
Source: J Patient Rep Outcomes. 2026 Jun 17;10:158. doi: 10.1186/s41687-026-01129-7 (PMC13526730; doi:10.1186/s41687-026-01129-7)
Supplement: Supplementary file 1 [file 41687_2026_1129_MOESM1_ESM.docx]

**Appendix 1: Sensitivity analysis**

Analysis 1A: Participants who reported no change in pain status between assessments

The following analysis restricts to participants who reported no change in pain status between assessments. This may provide a cleaner estimate of test-retest reliability when the underlying construct is stable.

Table 1**:** sensitivity analysis results restricted to participants who reported no change in pain status between assessments (n=34)

| **Outcome** | **N** | **ICC** | **95% CI** | **SEM** | **SDC** | **Mean diff** | **SD diff** |
| --- | --- | --- | --- | --- | --- | --- | --- |
| Pain-related Fear (0-16) | 34 | 0.68 | 0.44 to 0.82 | 2.33 | 6.45 | -0.41 | 3.33 |
| Pain-related Avoidance (0-24) | 34 | 0.65 | 0.41 to 0.81 | 3.92 | 10.87 | -1.50 | 5.45 |
| Pain Interference (0-48) | 34 | 0.66 | 0.42 to 0.81 | 6.63 | 18.38 | 0.04 | 9.57 |

Interpretation: Restricting to participants who reported no change in pain status resulted in ICCs of 0.68 for fear (vs 0.74 in main analysis), 0.65 for avoidance (vs 0.62), and 0.66 for interference (vs 0.71). These estimates are similar to the main analysis, with all ICCs remaining in the moderate range (0.5–0.75). This suggests that including participants who reported a change in pain status did not substantially bias the reliability estimates.

Limitations: This sensitivity analysis has several limitations. First, the sample size is reduced (N=34 for fear/avoidance, N=34 for interference), resulting in wider confidence intervals and less precise estimates. Second, the classification of 'no change' relies on participant self-report at the second assessment, which may not accurately capture true stability in the underlying pain construct. Third, participants who reported no change may differ systematically from those who reported change, limiting generalisability.

**Analysis 1B: Test-retest interval sensitivity analysis**

To examine whether variability in the test–retest interval influenced the reliability estimates, the association between interval length and score stability was assessed, and the ICC analyses were repeated for participants whose interval fell within the intended 2–4 week window (≤28 days).

Spearman correlations between interval length and the absolute between-assessment difference (|T2 − T1|) were weak and non-significant for pain-related avoidance (rho = 0.23, p = 0.14) and pain interference (rho = −0.07, p = 0.63), and weak for pain-related fear (rho = 0.30, p = 0.05). When the ICC analyses were restricted to participants with an interval of ≤28 days, estimates remained in the moderate range and were consistent with the main analysis: pain-related fear ICC = 0.74 (95% CI 0.53–0.86, n = 33), pain-related avoidance ICC = 0.69 (95% CI 0.47–0.84, n = 33), and pain interference ICC = 0.71 (95% CI 0.49–0.84, n = 34), with confidence intervals overlapping those of the main analysis. This indicates that variability in the test–retest interval did not materially influence the reliability results.
